# Draft genome sequence of a multidrug-resistant *Klebsiella pneumoniae* MTR-Kp-OBF01 strain isolated from an ornamental bird (*Melopsittacus undulatus*) in Bangladesh

**DOI:** 10.1128/mra.01214-25

**Published:** 2025-11-25

**Authors:** Md. Abdullah Evna Hasan, Sadia Afrin Punom, Md. Saiful Islam, Md. Liton Rana, Md. Tabeer Hossain Antor, Zuhayr Bakhtiyar, Naeem Ahammed Ibrahim Fahim, Dilruba Akter Jany, Mahbubul Pratik Siddique, Mohammad Ferdousur Rahman Khan, Md. Tanvir Rahman

**Affiliations:** 1 Department of Microbiology and Hygiene, Faculty of Veterinary Science, Bangladesh Agricultural University 54492, Mymensingh, Bangladesh; 2 Department of Animal Science, University of California – Davis 166631, Davis, California, USA; 3 National Engineering Research Center of Industrial Wastewater Detoxication and Resource Recovery, Research Center for Eco-Environmental Sciences, Chinese Academy of Sciences 385197, Beijing, China; 4 University of Chinese Academy of Sciences 74519, Beijing, China; University of Manitoba, Winnipeg, Manitoba, Canada

**Keywords:** whole-genome sequencing, *Klebsiella pneumoniae*, MDR, ornamental birds, Bangladesh

## Abstract

We report the draft genome sequence of a *Klebsiella pneumoniae* strain MTR-Kp-OBF01, isolated from a fecal sample of an ornamental bird from a local pet bird shop in Mymensingh city, with a total length of 4,854,485 bp, 1 plasmid, 24 predicted antimicrobial resistance genes, and 10 predicted virulence factor genes.

## ANNOUNCEMENT

Ornamental birds, particularly psittacines, are kept as companions but may harbor zoonotic bacteria of public health concern (1). As potential reservoirs of multidrug-resistant (MDR) pathogens, they pose transmission risks to humans and animals (2). Here, we present the draft genome sequence of a MDR *Klebsiella pneumoniae* isolated from an ornamental bird.

In January 2025, a freshly dropped fecal sample from an ornamental bird (budgerigar [*Melopsittacus undulatus*]) was collected aseptically from a local pet bird shop in Bridgemore, Mymensingh (24.7425°N, 90.4168°E), transferred to 5 mL of nutrient broth in the laboratory, and then incubated aerobically at 37°C overnight. The overnight culture was then streaked onto MacConkey Agar (HiMedia, India; catalog no. M081-500G) and incubated at 37°C overnight. A single lactose-fermenting colony (mucoid and pink to red) was selected and repeatedly subcultured until a pure culture was obtained. Phenotypic identification was performed based on colony morphology and Gram staining (3). The strain was identified as MDR through the Kirby-Bauer disk diffusion test, which revealed resistance to at least three antimicrobial classes (4). The bacterial isolate was then cultured in Luria-Bertani broth (BD, USA; catalog no. 244620), and DNA was extracted from the cultured broth using the DNeasy Blood & Tissue Kit (Qiagen, Germany; catalog no. 69504). Subsequently, the DNA library was prepared with the Illumina DNA Prep Reagent Kit (Illumina, USA; catalog no. 20060059), and sequencing was conducted on the Illumina NextSeq 2000 platform, generating paired-end reads with a 2 × 150 bp length. The genome assembly was performed using SPAdes v3.15.5 (5), and trimming of Illumina adapters was done using FastP v0.23.4 (6). Quality assessment was performed through FastQC v0.12.1(7), and genome annotation was performed using the NCBI Prokaryotic Genome Annotation Pipeline (PGAP) v.6.10 (8). Taxonomic classification and contamination screening were performed using Kraken2 v2.1.3 (9), and genome completeness was assessed with CheckM v1.2.3 (10). The sequence type was predicted using MLST v2.11 (https://github.com/tseemann/mlst), and virulence factor genes (VFGs) were identified using the virulence factor database (VFDB) (11) via ABRicate (https://github.com/tseemann/abricate). Moreover, the pathogenicity index, plasmids, antibiotic resistance genes (ARGs), bacteriocin gene clusters, prophages, and metabolic subsystems were predicted using PathogenFinder v2.0.0 (12), PlasmidFinder v2.1(13), the comprehensive antibiotic resistance database with RGI main v.6.0.2 (14), BAGEL4 (15), PHASTEST v1.0.1(16), and RAST v2.0 (17), respectively. Unless otherwise noted, default parameters were used for all software.

The genome characteristics of the *K. pneumoniae* MTR-Kp-OBF01 strain are given in Table 1. This genome comprises 4,854,485 bp with a GC content of 57.25%. *De novo* assembly produced 64 contigs (>500 bp) with an N50 of 303,930 bp. The genome exhibits an average nucleotide identity of 99.30% compared to the reference genomes of *K. pneumoniae*. The genome was identified as sequence type ST515 using seven-gene multilocus sequence typing (*gapA, infB, mdh, pgi, phoE, rpoB*, and *tonB*). Furthermore, the genome contained one plasmid [IncFIB(K)(pCAV1099-114)], 24 predicted ARGs, 10 predicted VFGs, 324 subsystems, 1 prophage, and 2 bacteriocins. The strain had a high predicted pathogenicity score of 0.914, matching 244 known pathogenic families, indicating substantial potential for virulence in both animals and humans.

**TABLE 1:** Genomic features of the *K. pneumoniae* strain MTR-Kp-OBF01 isolated from ornamental birds

Attributes	Values
BioProject accession	PRJNA1344620
GenBank accession	JBRQJS000000000
BioSample accession	SAMN52649697
SRA accession	SRR35775480
Assembled genome size (bp)	4,854,485 bp
Guanine–cytosine %	57.25%
Sequencing depth (×)	~88×
Genome completeness (%)	96.32%
Contamination	0.05%
N50 value (bp)	303930
Number of plasmids	1 [IncFIB(K)(pCAV1099-114), 99.82% identity (560 bp, contig NODE_34, positions 2187-2746; reference CP011596)]
Number of contigs	116
Genes (total)	4856
CDSs (total)	4750
Genes (coding)	4624
CDSs (with protein)	4624
Genes (RNA)	106
rRNAs	1, 9, 7 (5S, 16S, 23S)
Partial rRNAs	1, 9, 7 (5S, 16S, 23S)
tRNAs	74
ncRNAs	15
Pseudo genes (total)	126
CDSs (without protein)	126
Pseudo genes (ambiguous residues)	0
Pseudo genes (frameshifted)	61
Pseudo genes (incomplete)	74
Pseudo genes (internal stop)	22
Pseudo genes (multiple problems)	27
Number of ARGs	24
Number of VFGs	10
Number of subsystems	354
Pathogenicity score	0.914
Matched with pathogenic families	244
Average nucleotide identity (ANI) (%)	99.30%
Reference genome (RefSeq)	GCF_023546055.1_ASM2354605v1_genomic.fna
Number of intact prophages	1
Number of bacteriocins	2

## Data Availability

The whole genome shotgun (WGS) project of the K. pneumoniae strain MTR-Kp-OBF01 has been deposited in GenBank under the accession number JBRQJS000000000. The version described in this paper is JBRQJS000000000.1. Associated data, including the raw sequencing reads, are available under BioProject accession PRJNA1344620, BioSample accession SAMN52649697, and SRA accession SRR35775480.
